# The effect of kids yoga practices on attention levels and frontal EEG theta/beta ratio

**DOI:** 10.3389/fpsyg.2025.1650897

**Published:** 2025-10-10

**Authors:** Hande Baba Kaya, Levent Görün, Rumeysa Alper, Selahattin Akpınar, Mustafa Koç, Aylin Çelen, Yeşer Eroğlu Eskicioğlu, Şirin Pepe

**Affiliations:** ^1^Faculty of Sport Sciences, Duzce University, Duzce, Türkiye; ^2^Faculty of Education, Duzce University, Duzce, Türkiye; ^3^Faculty of Sport Sciences, Abant Izzet Baysal University, Bolu, Türkiye

**Keywords:** kid’s yoga, attention, EEG theta/beta ratio, volleyball athletes, cognitive performance

## Abstract

**Introduction:**

The aim of this study was to investigate the effects of children’s yoga practices on attention levels and frontal EEG theta/beta power ratios in young athletes.

**Methods:**

This research was conducted using a triple regulation design from mixed research methods. The participants were 20 female midi volleyball players (Mean = 11.45; SD = 0.49) with 3 years of volleyball experience from Düzce Volleyball Sports Club. They were randomly assigned to experimental and control groups. The experimental group participated in 24 sessions of children’s yoga, each lasting 40 minutes, at least 3 days per week for 8 weeks, including asanas (postures) and pranayama (breathing) techniques designed for children. The control group did not receive any intervention. Quantitative data were collected using the d2 attention test and Nexus 10 Mk II EEG device, while qualitative data were obtained through semi-structured interviews. Two-way repeated measures ANOVA was applied to the quantitative data, and thematic analysis was used for the qualitative data.

**Results:**

The d2 attention test results indicated significant improvements in TN-E scores for the experimental group compared to the control group (Wilks’ *λ* = 0.226, F(1,18) = 61.74, *p* < 0.05). EEG analysis showed significant effects of yoga on frontal theta/beta power ratios: Fz (Wilks’ *λ* = 0.987, F(1,18) = 19.32, *p* < 0.05, *η*^2^ = 0.518), F7 (Wilks’ *λ* = 0.692, F(1,18) = 8.01, *p* < 0.05, *η*^2^ = 0.308), and F8 (Wilks’ *λ* = 0.605, F(1,18) = 11.74, *p* < 0.05, *η*^2^ = 0.395). Qualitative findings indicated that coaches observed higher focus and better responses to strategies among athletes in the yoga group.

**Discussion:**

These findings suggest that children’s yoga practices have a significant impact on improving attention levels and frontal EEG theta/beta ratios, contributing to enhanced neurophysiological indicators of attention. Coaches also reported improvements in concentration and performance-related behaviors. Therefore, children’s yoga practices may be considered as a supportive training tool to enhance young athletes’ performance.

## 1 Introduction

Attentional control is a critical cognitive function that enables individuals to selectively focus on relevant stimuli while ignoring distractions. This ability is essential for a variety of higher-order cognitive processes, including learning, memory, and emotional regulation (Diamond, 2013; Shipstead et al., 2014). Inability to control attention is seen as a feature of important disorders affecting cognitive function in individuals (Eysenck et al., 2007; Paulus, 2015; Zainal and Newman, 2018; Kertz et al., 2016; Gagne et al., 2017). This important feature of attentional control requires measurement, but how to measure it most reliably is still a subject of research. Traditional methods have generally failed to produce consistent results. Therefore, innovative measurement tools are needed to accurately measure attentional control (Draheim et al., 2021).

The use of electroencephalography (EEG) to measure attentional control has become increasingly common in recent years. Research has pointed to the potential of spontaneous EEG to reflect attentional control processes without having to rely on specific measurement parameters (Wei et al., 2024). This approach suggests that the natural electrical activity of the brain provides important clues about individuals’ attentional processes (Putman et al., 2010). Electroencephalographic (EEG) signals reflect electrical fluctuations of brain activity (Nunez and Srinivasan, 2006). By monitoring the activity of brain waves, EEG provides information about individuals’ attention and mental focus levels. EEG signals can be analyzed by separating them between different frequency bands. Especially the ratio between theta (4–7 Hz) and beta (13–30 Hz) waves measured under resting conditions, the theta/beta ratio, provides important information about attention (Tzong-Shi et al., 2003; Pineda, 2005). The ratio of theta and beta waves provides important clues about the attention status and cognitive functions of individuals. Theta waves are associated with deep thinking, creativity and meditation state (Sürmeli, 2010) relaxation, introspection and creativity (Picken et al., 2019), while beta waves are more associated with attention, focus (Sürmeli, 2010), alertness and mental processing (Picken et al., 2019). In this context, theta/beta ratio analysis can be used as a useful marker for evaluating important clues about individuals’ cognitive processes (Cavanagh and Frank, 2014). It is suggested that attention control is controlled by two reciprocal systems. These are subcortical areas (amygdala, thalamus, pre singulate cortex) associated with the reception of stimuli and initiation of interaction (Bishop, 2008; Hermans et al., 2014) and the lateral prefrontal cortex mediating the task of maintaining attention (Fani et al., 2012; Gregoriou et al., 2014; Miller and Cohen, 2001). Anterior-frontal EEG measurements from the region that mediates attention maintenance and attentional control allow accurate monitoring of attention-related processes, as these processes directly reflect electrical activity in this region of the brain. Since the pre-frontal region plays a critical role especially in cognitive control and attention processes, EEG data obtained from this region provide high accuracy in understanding attention management. Studies show that there is an increase in the theta/beta ratio in individuals with attention deficit (Arns et al., 2013). In healthy individuals, an inverse relationship was found between stress-induced attention deficit, attentional orientation and attentional control and theta/beta ratio (Putman et al., 2010; Morillas-Romero et al., 2015; Angelidis et al., 2016). In Putman et al.’s studies, there is evidence that the theta/beta ratio may reflect attentional control in healthy individuals and that the frontal theta/beta ratio is related to executive control functions (Putman et al., 2010; Putman et al., 2014). Barry, Clarke, and Johnstone (Barry et al., 2003) stated that the increase in theta waves is related to attention and cognitive load and that high theta/beta ratio may lead to distraction and decreased cognitive performance. These findings reveal that the theta/beta ratio is an important electrophysiological marker that allows interpretation on attention control (Angelidis et al., 2016). This method allows direct and objective assessment of individuals’ attention levels.

In fast-paced and strategy-demanding branches in sports environments, lack of attention control can lead to negative consequences such as not being able to intervene in time, taking the wrong position and making strategic mistakes. Volleyball is a team sport that requires fast reflexes, strategic thinking and high attention. Although there are many factors affecting the performance of volleyball players, the level of attention control stands out as one of the most critical elements of success (Sheppard et al., 2009; Savelsbergh et al., 2002). The quality of athletes’ performance depends on many factors such as neuro motor abilities, effective cortical control, mental and motor memory, coordination, and attention (Gorman et al., 2015). In recent years, deficits in attention control have become a wide-spread problem among athletes. In this context, it is important to examine the effects on attention levels and neurophysiological responses of volleyball players in order to improve their performance. For this reason, various alternative methods have been adopted to improve athletes’ attention control. Among these methods, kids yoga attracts attention. Yoga is an inner journey that includes experience (Osho, 2022). Patanjali defined yoga in the Yoga Sutras as taking mind waves under control (Venkateswarlu and Begum, 2023). The main goal of yoga is to integrate consciousness (Büssing et al., 2012). Yoga positively affects cognitive functions such as attention by increasing concentration and focus with the pranayama (breathing techniques) and asana (poses) practices it contains (Nanthakumar, 2018; Hagen and Nayar, 2014; Eggleston, 2015). Yoga, which develops breath control and body awareness, stands out as an alternative practice that has positive effects on attention, motor skills, stress and anxiety (Nanthakumar, 2018; Manjunath and Telles, 2004; Gulati et al., 2019; Hagen and Nayar, 2014; Holt, 2016; Berger et al., 2009; Eggleston, 2015; James-Palmer et al., 2020; Lin et al., 2015; Nilsoge et al., 2016; Ghosh, 2023; Sibalis, 2017). Studies have shown that yoga practice provides better attention control by promoting focused attention and relaxation that can help balance theta and beta activities (Lin et al., 2015; Singh and Mutreja, 2020) and that regular yoga activities support kids emotional and psychosocial well-being and help them become mentally, spiritually and physically healthier (Bazzano et al., 2018; Peker and Olgan, 2022; Telles et al., 2013; Eggleston, 2015; Hariprasad et al., 2013; James-Palmer et al., 2020).

In summary, the present study was conducted to examine the effects of kids yoga practices on the attention control levels of volleyball players. The findings of the study provide important data about the usability of yoga practices as a new strategy that can be applied to increase attention control. The following hypotheses are proposed within the scope of this study.

*H1:* “Participation in kids yoga practices will significantly improve attention scores in volleyball players.”

*H2:* “Participation in kids yoga practices will result in a significant reduction in the frontal EEG theta/beta ratio.”

## 2 Method

### 2.1 Research model

This research was conducted using the combined design, which is one of the mixed research methods. The aim of this design is to explain similarities or differences by combining separately collected qualitative and quantitative results (Creswell, 2021). In the first stage of the mixed method research, qualitative data based on the coaches’ opinions on the attention levels of volleyball players were collected; in the second stage, quantitative data were obtained by taking the d2 attention test and frontal EEG recordings to determine the attention levels of the athletes. Participants were randomly assigned to the experimental and control groups using a computer-generated random number table. After random assignment, baseline equivalence between groups was assessed for pre-test scores. The in-dependent variable, children’s yoga treatments, was administered to the experimental group, while no treatment was administered to the control group. In this model, both pre-test and post-test data were collected from the experimental and control groups, and the effect of children’s yoga treatments was examined (Karasar, 2023; Büyüköztürk, 2014). After the experimental process, individual interviews were conducted with the coaches to evaluate the changes in the experimental group from the coach’s perspective. The qualitative and quantitative data were combined and interpreted.

### 2.2 Participants

A total of 20 participants (*n* = 20; Mage = 11.45; SD = 0.49) playing in the Duzce Volleyball Youth and Sports Club girls’ midi volleyball team were invited to participate in the study. Participants had 3 years of volleyball experience. Written consent was obtained from the families of all participants before the experimental application and they were informed that they had the right to withdraw from the research at any time. In the qualitative dimension of the research; 2 coaches (C1, C2) working in the Duzce Volleyball Sports Club Midi category were included in the study.

### 2.3 Data collection

#### 2.3.1 d2 attention test

**Figure 1:**
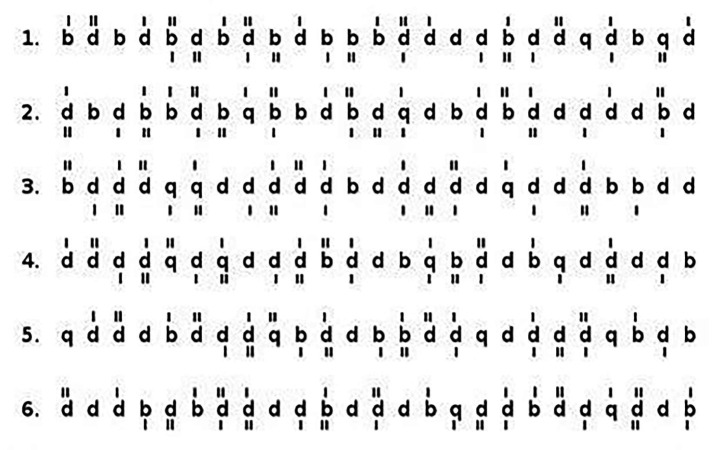
Sample presentation of d2 attention test.

This test was developed by Brickenkamp in 1962 and can be applied to individuals between the ages of 9–60. There are 14 lines in the test form and 47 numbers in each line. There is a 20-s time limit for each line of the 14-line test. The test includes letters with 1, 2, 3 or 4 dots above or below them. In this test, letters can be found in 16 different ways depending on their number, the points they receive and their order. Participants are in-structed to identify the letter ‘d’ with exactly two dots, among similar distractor items. It was developed to measure the individual’s attention level, psychomotor speed, learning functions, selective attention and mental concentration. The test is evaluated using TN (total number of marked lines), E1 (number of digits skipped without being marked), E2 (number of letters marked incorrectly), CP (total number of marked lines), TN-E (test performance) and E% (error rate) scores. In this study, the TN-E score, which gives the total performance value of the test, was used. The reason for including the TN-E score, which represents the balance between psychomotor speed and selective attention, in the study is that the attention levels in the test battery are interpreted by looking at this score. In interpreting the test, the TN-E score calculations were made by following the instructions in the test battery (see Figure 1).

#### 2.3.2 Nexus 10 Mk II neurofeedback/biofeedback and physiological imaging, evolution and feedback system

**Figure 2:**
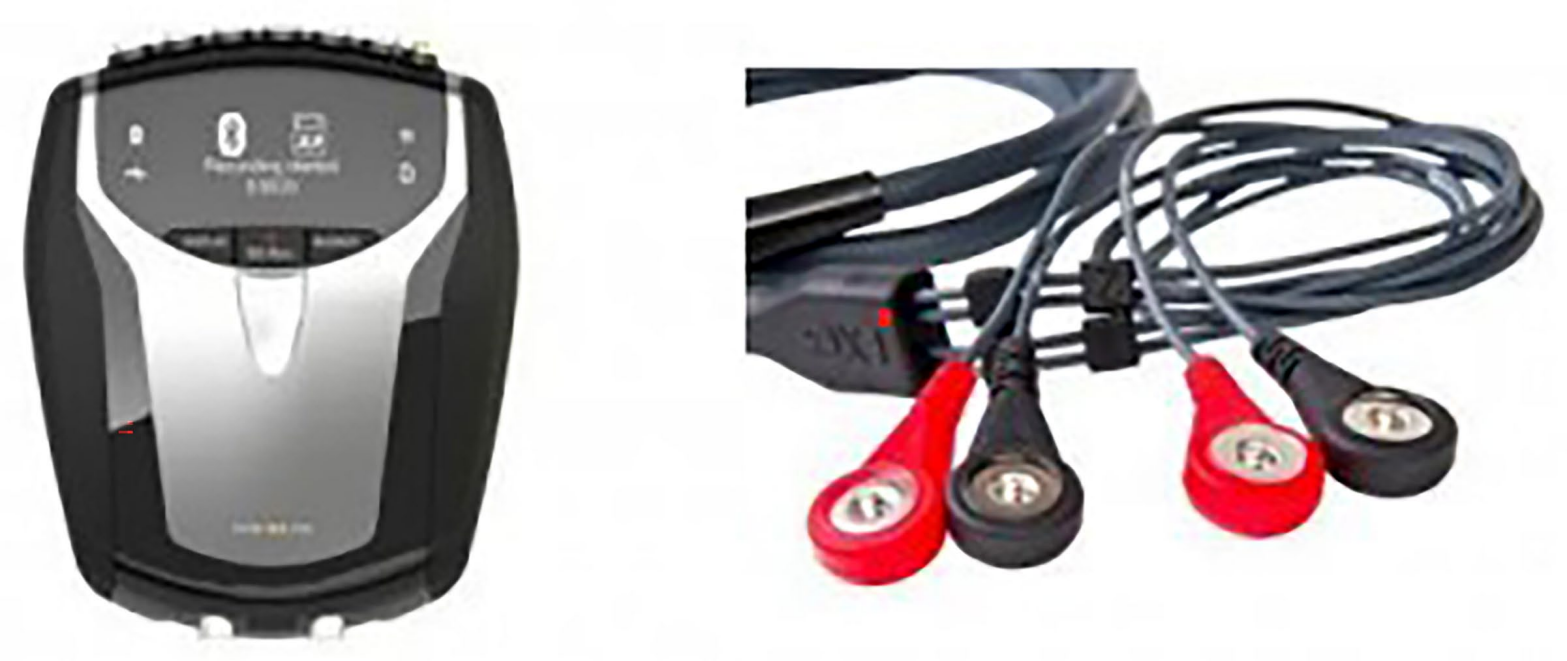
Nexus 10 Mk II device image.

Within the scope of the study, EEG recordings were recorded with the Nexus 10 Mk II device as a neurophysiological marker to determine the attention levels of the athletes. When we look at the technical specifications of the device; EXG bandwidth DC-2KHz (ultra-wideband), EXG sampling rate 8,192 samples/s (max.), EXG dynamic range ±200,000 μV (pk-pk). The NeXus-10 Mark II records all EEG signals in a single recording. The system software records the activity of the whole brain and can display multiple analyses and components at the same time by applying different filters. The software offers all standardized waves such as Alpha, Beta, Delta, Gamma, Gamma, Theta, SMR and can be adjusted according to each frequency range when necessary, and also establishes mathematical and statistical relationships between these waves. For example, it provides data such as Theta/Beta, Alpha/Theta and their FFT data. The device uses BioTrace+ soft-ware. It enables the reduction of artefacts and noise when recording physiological signals at microvolt levels. The NeXus 10 Mk II uses automatic artifact removal algorithms in its software to eliminate involuntary signals such as eye blinks and muscle movements (NeXus 10 Mk II User Manuel). This is essential to obtain clean data, which is especially important when analysing EEG signals. The software automatically filters out noise and artifacts by measuring them and removing them from the total signal, so that the remaining signal is the real signal, free of noise and clean. With the help of sensors connected to the device, recording is made to the computer environment. During the recording, the practitioner can view the recorded data with the help of dual monitors (Mirifar et al., 2019). In the literature, it is seen that studies have been conducted using the NeXus 10 Mk II device. These studies show the effectiveness and usability of the device (Mirifar et al., 2019) and detailed information on artifact removal is included in the software and user manual of the device (NeXus 10 Mk II User Manual^1^; BioTrace+ software User Manual^2^) (see Figure 2).

#### 2.3.3 Experimental procedure

After the participants’ preliminary measurements were taken, the experimental group was informed about the gym where the yoga practices would be held and a class schedule was created by determining the days they were available for participation.

Participants were asked to participate in practices lasting less than 40 min, at least 3 days a week for 8 weeks, for a total of 24 sessions.

Sample one-session children’s yoga program:

- Greetings and roll call: 3 min.
- Pranayama (10 fingers, 10 breaths): Get into a comfortable sitting position. Place your hands on your legs. Now bring your attention to the breaths coming in and out through your nose. Close one of your fingers after each exhalation. When the 10 breaths are over and both hands are clenched into its, start opening one of the fingers after each exhalation. Continue until the fingers of both hands are fully open: 2–3 min.
- Warm-up exercises to improve attention (Video watching): 5 min.
- Asanas: Sun Salutations and Shavasana (Corpse pose): 25 min.
- Activity card: 4 min.

In the kids yoga program, asanas (poses) and pranayama (breathing) techniques developed for children were used. The sessions ended with kids yoga games to support attention development. The applications were carried out by an expert who has received training in children’s yoga and holds an internationally valid certificate. The study was terminated after the post-measurements were taken.

After the pre-test measurements taken from participants randomly assigned to the control group, no action was taken within the scope of the research. They continued their normal lives during the 8-week application period. Post-test measurements were taken when the application process was completed.

#### 2.3.4 Quantitative data collection

Data collection was scheduled on separate days to avoid potential mental fatigue from the d2 test interfering with the EEG results Therefore, to ensure the accuracy and reliability of the data, the independent and consistent data collection process was guaranteed.

##### 2.3.4.1 Application of d2 attention test

The d2 attention test, which was used as a data collection tool within the scope of the re-search, was applied individually in a quiet environment based on the voluntary participation of the participants. Measurements lasted 5–8 min on average for each child. d2 attention test measurements were collected from children at the same time of the day. All d2 test sessions were scheduled at the same time of day (midday) to minimize the influence of circadian fluctuations on cognitive performance (Monk, 2005).

##### 2.3.4.2 EEG data collection

**Figure 3:**
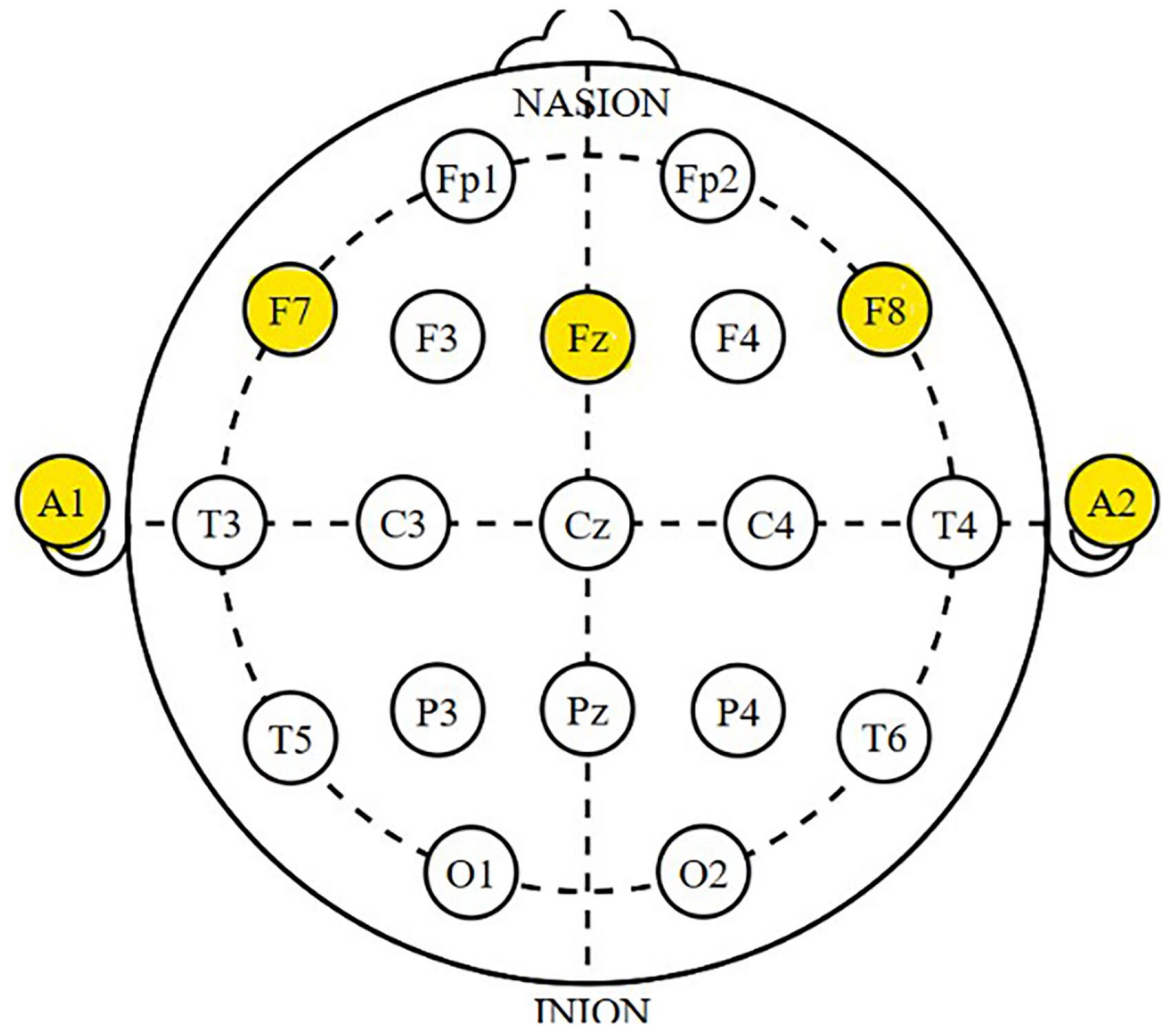
Electrode locations of international 10–20 system for encephalography recording (Jasper, 1958).

For recording, the standard electrode placement point published by Jasper in 1958 and referred to in the literature as the international 10–20 system was used (Figure 3). Electroencephalogram (EEG) recording was taken from the frontal region; Fz, F7 and F8 electrode sites. While taking theta/beta recordings, the electrode was placed at the Fz point (F7 and F8 points, respectively) and the reference electrodes were placed on both earlobes (A1 and A2). The impedance was kept below 5 kΩ. Fz (Frontal Z) is located in the middle of the frontal lobe and is associated with cognitive processes, attention, emotional responses and motor control. EEG measurements at Fz generally follow theta and beta waves. F7 (Frontal 7) is close to the left frontal region and is related to cognitive processing, attention, memory and social cognitive functions. F7 is generally sensitive to alpha and theta waves. F8 (Frontal 8) is close to the right frontal region and is associated with emotional responses, cognitive control and attention. F8 generally follows theta and beta waves (Niedermeyer and Da Silva, 2004; Buzsáki and Draguhn, 2004; Cohen, 2017; Hughes and John, 1999). When previous studies are examined, it is seen that neural activity in the frontal region plays different roles in the attention control process (Putman et al., 2010, 2014; Angelidis et al., 2016; Angelidis et al., 2018; Van Son et al., 2018; Nitsche et al., 2012; Sari et al., 2016; Monk, 2005; Morillas-Romero et al., 2015). Ten-minute recordings were taken from each region (Fz, F7 and F8). According to the general EEG literature, it is generally recommended that the recording duration varies between 5–10 min in studies examining attention and cognitive processes (Posner and Petersen, 1990).

##### 2.3.4.3 EEG signals

In this study, EEG data were collected in raw form and analysed by the Root Mean Square (RMS) method. RMS is calculated by taking the square root of the mean of the squares of the amplitude value of the signal and is generally used as a common method for power analysis of EEG data (Niedermeyer and Da Silva, 2004). This calculation was per-formed with a sampling rate of 256 SPS (samples per second). The power spectrum was calculated using the Fast Fourier Transform (FFT). FFT is a widely used method for analysing frequency components and is a standard technique for frequency analysis of EEG signals (Buzsáki, 2006). The FFT frequency range was set to 60 Hz. The epoch size was set to 1/4 refresh time (0.250 Hz) for each analysis window, which usually allows monitoring rapid changes in the EEG signal (Vanhatalo and Kaila, 2006). The peak-to-peak method was used to measure the EEG signal. This method determines the total signal amplitude by measuring the difference between the maximum and minimum amplitude values of the signal (Srinivasan et al., 2012). The automatic scaling option (Auto μV Range) was select-ed for minimum and maximum values per brain map, and a fixed scale (2–45 μV peak-to-peak) was also selected for all brain maps. In this study, theta (4–8 Hz) and beta (13–21 Hz) band power values of EEG were automatically calculated and averaged. The-ta/beta ratio is frequently used in the literature as an important parameter related to attention and cognitive processes (Cavanagh et al., 2011). In the spectral analysis, the power density in theta and beta bands was calculated at specific frequency ranges. This calculation was performed with a sampling rate of 32 SPS (samples per second). Theta band power is defined as 4–8 Hz and beta band power as 13–21 Hz, and these frequencies are generally associated with attention control and alertness (Laufs et al., 2003). The epoch size used in the analyses was determined as 2 s. This epoch length is ideal to ensure that the signal is examined at sufficient time intervals and temporal changes are observed (Nitsche et al., 2012).

#### 2.3.5 Qualitative data collection

During the qualitative data collection phase of the study, semi-structured questions were prepared, and individual interviews were conducted with coaches. During the inter-views, the coaches’ opinions on the distracting factors observed in athletes and how they managed these situations were collected. After the study, the coaches were asked whether the kids yoga practice led to any changes in the athletes’ attention levels, and their responses were recorded. The two coaches included in the data collection process are individuals who have been working with the athletes for a long time and are experienced in observing the effects of attention levels on training performance. Both coaches have pedagogical formation and have previously received training on the concept of attention in sports. In this respect, they were evaluated as reliable data providers in terms of both their contextual knowledge and observational experience. The interview form was developed by obtaining expert opinions (reviewed by three experts with at least a doctorate degree in sports sciences) and evaluated in terms of conceptual validity. A trial interview was con-ducted to verify the comprehensibility of the questions. Necessary revisions were made in line with the suggestions of the experts and the form was finalized. Below are the questions used in the interview. Semi-structured interview questions pre-measurement;

1. Do you often encounter athletes being distracted during training and matches? In what situations is this more evident? Are there certain behaviours or signs?
2. Semi-structured interview questions were the final measurement;
3. Have you observed any changes in the attention levels of your athletes during training and matches after kids yoga practice?
4. What do you think about the effects of kids yoga practices on attention levels in the long term?

### 2.4 Analytical strategy

#### 2.4.1 Analysis of qualitative data

Thematic analysis was used in the analysis of interviews with coaches and a deductive approach was adopted (Braun and Clarke, 2023; Finlay, 2021). Therefore, the stages of determining design issues and final themes, verification and use of the code were followed with a gradual coding and recoding process. In order to ensure the reliability of the re-search, the data were coded independently by 4 researchers who are experts in the field (Ekiz, 2020). In addition, the data collected under the themes determined by the researchers conducting the study were compared with two experts outside the study to reveal the numbers of agreements and disagreements, and then the reliability of the study was calculated using the Miles and Huberman (1994) formula.

#### 2.4.2 Analysis of quantitative data

All data are presented as mean ± SD unless otherwise stated. All statistical analyses were completed using SPSS Statistics, version 27 (IBM, NY, USA). Data were tested for normality. Pre–Post test data were analysed using two-way ANOVA for mixed measurements. When the sphericity assumption was violated, the Greenhouse–Geisser correction was employed. Pairwise comparisons were assessed using the Bonferroni correction. Significance was set at 0.05 (2-tailed) for all analyses. The effect sizes obtained in the study were reported using partial eta squared (*η*^2^) values. These effect sizes were interpreted based on the classification proposed by Cohen (1988) as follows: *η*^2^ < 0.01, negligible effect; 0.01 ≤ *η*^2^ < 0.06, small effect; 0.06 ≤ *η*^2^ < 0.14, medium effect; *η*^2^ ≥ 0.14, large effect. This classification is meaningful in terms of indicating the proportion of variance explained. The obtained *η*^2^ values were interpreted by comparing them to these thresholds (Cohen, 1988).

## 3 Findings

### 3.1 Analysis of quantitative data

As shown in Table 1, the mean scores of theta/beta power ratios of the experimental group participating in kids yoga practices before the practice were Fz Mean = 5.34, F7 Mean = 4.32; F8 Mean = 4.74, while this value became Fz Mean = 3.71, F7 Mean = 3.35, F8 Mean = 3.41 after the practice. The mean scores of theta/beta power ratios of the control group, which did not receive any practice, in the pre-measurements were Fz Mean = 6.18, F7 Mean = 5.340; F8 Mean = 4.89, while this value became Fz Mean = 7.48, F7 Mean = 6.63, F8 Mean = 5.92 in the post-measurements. Considering the inverse relationship between theta/beta power ratio of the frontal region and attention level, the decrease in the pre-measurement scores of the experimental group in the post-measurement can be interpreted as an increase in attention control levels. In addition, while the TN-E value obtained from the d2 attention test before the application of the experimental group participating in kids yoga practices was 335.60; this value became 579.00 in the final measurement. While the TN-E value of the control group was 312.90 in the pre-measurement; this value became 313.80 in the final measurement. Accordingly, it is seen that there was an increase in the d2 attention test TN-E value in the post-test scores of the experimental group and there was no change in the control group.

**Table 1:** The mean (M) and standard deviation (SD) of EEG indicators: theta/beta power ratio and attentional test (TN-E).

Measurement		Theta/beta power ratio						Attentional control measures	
		Experimental groups			Control groups			Experimental groups	Control groups
		Fz	F7	F8	Fz	F7	F8	TN-E	TN-E
Pre-test	M	5.340	4.323	4.741	6.184	5.340	4.896	335.600	312.900
	SD	1.679	1.040	1.228	1.882	2.513	0.929	99.392	49.818
Post-test	M	3.715	3.355	3.412	7.482	6.634	5.923	579.000	313.800
	SD	.961	.603	.646	2.241	3.268	2.104	45.813	49.870

The two-factor ANOVA results regarding whether the changes observed after the application compared to before the application in the experimental group that received kids yoga applications and the control group that received no application showed a significant difference are given in Tables 2, 3.

**Table 2:** ANOVA results of the pre-test and post-test scores of frontal theta/beta power ratio.

Source of variation	Fz				F7				F8			
	df	F	*p*	*η* ^2^	df	F	*p*	*η* ^2^	df	F	*p*	*η* ^2^
Between subjects effects												
Group (experimental/control)	1	10.52	0.005	0.369	1	6.04	0.024	0.252	1	7.31	0.014	0.289
Error	18				18				18			
Within subjects effects												
Measure (pre/post-test)	1	0.242	0.629	0.013	1	0.167	0.688	0.009	1	0.193	0.666	0.011
Group*Measure	1	19.32	0.000	0.518	1	8.01	0.011	0.308	1	11.74	0.003	0.395
Error	18				18				18			

**Table 3:** ANOVA results of the pre-test and post-test scores of attention measures (TN-E).

Source of variation	TN-E			
	df	F	*p*	*η* ^2^
Between subjects effects				
Group (experimental/ control)	1	34.012	0.000	0.654
Error	18			
Within subjects effects				
Measure (pre/post-test)	1	62.666	0.000	0.777
Group*Measure (pre/post-test)	1	61.746	0.000	0.774
Error	18			

As shown in Table 2, it can be said that there was a significant difference between the pre-test and post-test scores in the frontal theta/beta power ratios of the athletes who participated in the experimental and control groups after the kids yoga applications, in other words, it can be said that the procedures applied in different groups created significant differences in the Fz, F7 and F8 electrode regions. In the Fz electrode region: Wilks’ *λ*: 0.987, *F*(1,18) = 19.32, *p* < 0.05, the significant difference seen (*η*^2^ = 0.518) has a large effect size; In the F7 electrode region: Wilks’ λ: 0.692, F(1,18) = 8.01, *p* < 0.05, the significant difference seen (*η*^2^ = 0.308) has a large effect size; In the F8 electrode region: Wilks’ λ: 0.605, F(1,18) = 11.74, *p* < 0.05, the significant difference seen (*η*^2^ = 0.395) has a large effect size. This finding shows that the kids yoga practice has an effect on the participants’ frontal theta/beta power ratios. In order to determine the source of the significant difference between the groups, the “Bonferonni compatible multiple comparisons” test was applied (Table 4). Furthermore, in order to control the potential Type I error risk arising from multiple comparisons regarding the significance of the group*measurement interaction, the Holm-Bonferroni correction was applied. Within the scope of this correction, the *p*-values were ranked in ascending order (Fz = 0.000, F8 = 0.003, F7 = 0.011) and each was compared with its corresponding threshold value calculated according to the Holm-Bonferroni method (Fz: 0.05 / 3 = 0.0167; F8: 0.05 / 2 = 0.025; F7: 0.05 / 1 = 0.05). As a result of these comparisons, all three *p*-values were found to be below their respective thresholds (Fz = 0.000 < 0.0167; F8 = 0.003 < 0.025; F7 = 0.011 < 0.05), and thus remained statistically significant after correction. These findings indicate that the changes observed over time in the EEG measurements of the experimental group (at Fz, F7, and F8 sites) were statistically significant compared to the control group. In other words, it can be concluded that the experimental intervention led to statistically significant and re-liable effects on EEG activity.

**Table 4:** *Post-hoc* pairwise comparisons (Bonferroni-adjusted) of pre-test and post-test frontal theta/beta power ratio scores between experimental and control groups.

Measurement		Mean Difference (experimental–control group)	SD	*p*
Fz electrode site	Pre-test	−0.844	0.798	0.304
	Post-test	−3.767*	0.771	0.000
F7 electrode site	Pre-test	−1.017	0.860	0.252
	Post-test	−3.279*	1.051	0.006
F8 electrode site	Pre-test	−0.155	0.487	0.754
	Post-test	−2.511*	0.696	0.002
TN-E	Pre-test	22.700	35.158	0.527
	Post-test	265.200*	21.415	0.000

*p < 0.05.

As shown in Table 3, it is seen that there is a significant difference (Wilks’ λ: 0.226, F(1,18) = 61.74, *p* < 0.05) between the pre-test and post-test scores of the d2 attention test TN-E value of the athletes participating in the experimental and control groups after the kids yoga practices. When we examine the common effect created by the groups and measurements, it can be said that different applications made on the groups create a difference in TN-E values and the eta squared statistic value of this common effect (*η*^2^ = 0.774) has a strong effect size. In order to determine the source of the significant difference between the groups, the “Bonferonni compatible multiple comparisons” test was applied (Table 4).

When the results of the Bonferonni compatible pairwise comparison analysis per-formed to see which groups the significant difference in frontal theta/beta power ratios in the Fz, F7 and F8 electrode placement regions between the experimental and control groups came from was examined, it was seen that there was no significant difference between the control group pre-test mean scores (Fz Mean = 6.18; F7 Mean = 5.34; F8 Mean = 4.89) and the experimental group pre-test mean scores (Fz Mean = 5.34; F7 Mean = 4.32; F8 Mean = 4.74). The absence of significant difference in the pre-tests proves that the groups were distributed homogeneously before the experimental procedure. It was observed that there was a significant difference between the control group post-test mean scores (Fz Mean = 7.48; F7 Mean = 6.63; F8 Mean = 5.92) and the experimental group post-test mean scores (Fz Mean = 3.71; F7 Mean = 3.35; F8 Mean = 3.41). This finding can be interpreted as kids yoga practices having positive contributions on attention levels by decreasing theta/beta power ratio in the frontal region. When the results of Bonferonni compatible paired comparison analysis conducted to see which groups caused the significant difference in d2 attention test TN-E value between experimental and control groups are examined, it is seen that there is a significant difference between control group post-test mean scores (M = 313.80) and experimental group post-test mean scores (M = 579.00).

**Figure 4:**
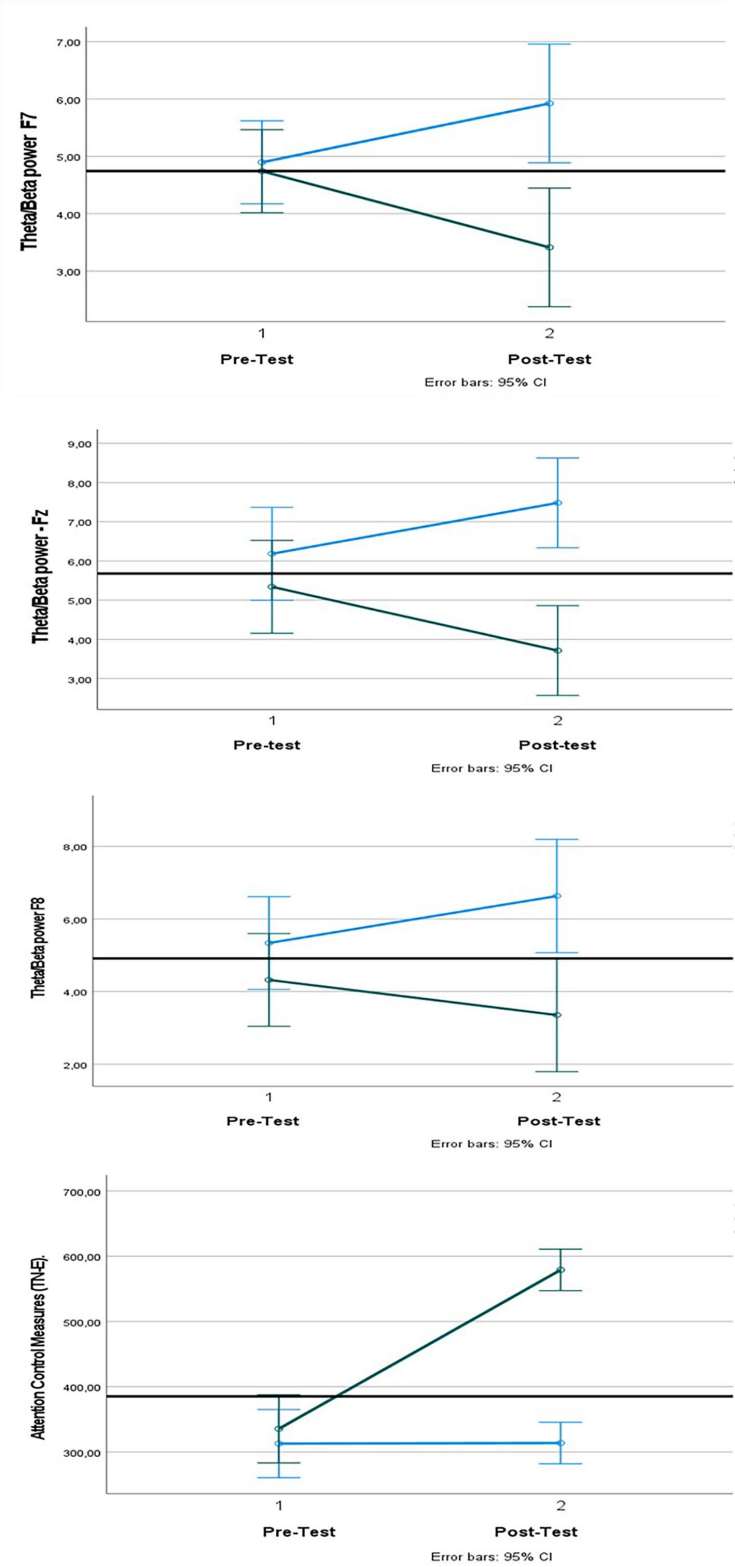
Pre- and post-test assessment of EEG indicators: theta/beta power ratio (for Fz, F7, F8 electrode site) and attentional measures (TN-E). Hz, hertz. Values displayed as mean + SD.

Figure 4 shows the comparison of the pre-test and post-test results of the frontal EEG theta/beta power ratios and d2 attention test TN-E values for the Fz, F7 and F8 electrode site of the experimental and control groups. It is seen that the frontal EEG theta/beta ratios of the experimental group participating in the eight-week yoga program decreased significantly compared to the control group, and the TN-E value increased significantly. This result can be interpreted as the yoga practices increased the attention levels of the experimental group participants and the balance between their psychomotor speed and selective attention increased. In this sense, it has been observed that kids yoga practices contribute positively to cognitive functions. The formula Δ% = [(pretest−posttest)/pretest]×100 was used to calculate the change between the pre-test and post-test scores of the kids yoga practices applied to the experimental group. It is seen that there is a change between the pre- test and post-test measurements of the experimental group for Fz electrode site as Δ% = 30.52, for F7 electrode site as Δ% = 22.45, for F8 electrode site as Δ% = 28.05, and for TN-E value as Δ% = 72.52.

### 3.2 Analyzing qualitative data

Table 5 presents the main themes and sub-themes from the coaches’ pre-measurement interviews. Two main themes were identified: ‘Factors causing distraction’ and ‘Observed dimension of distraction’ and seven sub-themes were identified: ‘Training Period’, ‘Match Period’, ‘Emotion and Motivation’, ‘Symptoms’ and ‘Signs’. Factors leading to distraction are listed as fatigue, monotony, personal problems, long training sessions and boring repetitions during the training period. During the match period, opponent performance, spectator effect, result pressure and individual mistakes contribute to distraction. Emotional effects, stress, anxiety, low motivation and internal pressures are also important factors in terms of emotions and motivation. Distraction can manifest itself with symptoms such as decreased concentration, poor communication and inattention, as well as signs such as not making eye contact and careless movements.

**Table 5:** Coaches’ opinions on the causes of distractions observed in the midi-volleyball girls’ team (pre-measurement).

Theme	Sub-theme	Coding
Factors causing distraction	Training period	Fatigue
		Monotony
		Personal problems
		Long training sessions
		Boring repetitions
	Match period	Opponent team performance
		Spectator effect
		Result pressure
		Individual mistakes
	Emotion and motivation	Emotional effects
		Stress and anxiety
		Effect of low motivation
		Internal pressures
Observed dimension of distraction	Symptoms	Decreased concentration
		Poor communication—lack of communication
		Inattention-difficulty in performing tasks
		Careless movements of athletes during training
	Signs	Not making eye contact
		Frequent glances at their watches
		Frequent diversion of attention

Table 6 presents the main themes and sub-themes obtained from the final measurement interviews of the coaches. Coaches stated that kids yoga practices had positive effects on athletes’ attention levels. They observed that the athletes were more focused, responded better to strategies and had less distractions after yoga. He also stated that yoga practices improved intra-team communication and co-operation by increasing stress coping, mental endurance and attention span.

**Table 6:** Coaches’ opinions on the effect of kids yoga on attention development in midi-volleyball girls’ team (post-measurement).

Theme	Sub-theme	Coding
The effect of kids yoga on attention development	Short-term attention increase	Increase in focus during training
		Maintaining attention during the match
		Improvement in quick decision-making skills
		Strengthening decision-making ability in critical moments
	Long-term performance development	Continuous attention enhancement
		Increase in performance in the game
		Strengthening mental endurance
	Mental relaxation and endurance	Reduction of stress
		Mental peace
		Emotional balance development
	Communication and cooperation within the team	Clarity and clarity in communication
		Common strategy development
		Increased team cohesion

In this direction, under the main theme of ‘The Effect of kids Yoga on Attention Development’, sub-themes such as short-term attention increase, long-term performance improvement, mental relaxation and endurance, intra-team communication and co-operation were formed. While short-term attention increase includes elements such as focusing during the moment-training, attention continuity in the match, and quick decision-making skills, long-term performance development refers to mental endurance and in-game performance increase. Mental relaxation and resilience include stress reduction and improving emotional balance, while clarity and co-operation in team communication are emphasized.

## 4 Discussion

The findings of this study show that 8 weeks of kids yoga interventions caused a significant increase in the attention levels of volleyball players in the experimental group. Comparisons with the control group revealed that this increase was statistically significant. This result is consistent with previous studies in the literature supporting the positive effects of kids yoga on attention development. Khalsa and Butzer (2016) reported that yoga practice increased children’s attention span and cognitive flexibility. Similarly, Metri (2018) found that children who followed a school-based yoga program showed significantly less anxiety and perceived stress compared to children who did not practice yoga. Petsche (2016) suggests that yoga can be used as a school-based intervention to improve the levels of task-oriented behavior of students with attention deficits. These findings support the potential of yoga practices to improve children’s attention levels. Attention is not only a cognitive process, but also related to emotional states. Brown and Ryan (2003) demonstrated the positive effects of mindfulness practices on attention and attention control.

The findings of the study are remarkable from a neurophysiological point of view. In the experimental group, 8 weeks of kids yoga led to a significant decrease in the frontal theta/beta power ratios of the participants. The change in frontal theta/beta ratios is associated with distractibility and it can be said that kids yoga has positive effects on attention by reducing this ratio. While frontal lobe is associated with attention and cognitive control, theta waves are associated with relaxation and distraction, and beta waves are associated with high attention and cognitive activities (Niedermeyer and Da Silva, 2004). In this context, kids yoga practices seem to be effective in increasing mental and physical aware-ness and attention balance (Kabat-Zinn, 2005). The increase in the theta/beta ratio observed in the control group may indicate an increase in distraction. Since the study was conducted during the summer (holiday) period, factors such as the absence of a school or sports program to keep children’s attention active, increased screen usage time during holiday periods, decreased physical activity levels and decreased educational activities that improve attention may have negatively affected the attention levels of the control group (Barutchu et al., 2019; Malatras et al., 2016; Turoman et al., 2021).

The relationship between yoga and EEG measurements, especially theta/beta ratio, is important. High theta/beta ratios indicate poor attention and executive function (Halawa et al., 2017). Studies have reported a decrease in the theta/beta ratio in healthy individuals after yoga practice. The group practicing yoga has been observed to have significant improvements in various cognitive functions such as increased performance, neural activity, attention and executive function (Nagendra et al., 2015). Another study indicating an in-crease in frontal theta/beta in states of mind wandering has shown that the decrease in the theta/beta ratio provides significant improvement in mind wandering attacks (van Son et al., 2019). Yoga may lead to changes in brain wave activity by promoting relaxation and reducing stress. Pal et al. stated that yoga practices can elevate alpha and beta activities by increasing parasympathetic state (Pal et al., 2014). Furthermore, Gaur et al. (2020) emphasized that yoga practices are linked to changes in brain wave dynamics, increased relaxation and improved cognitive function.

In summary, it appears that kids yoga has a beneficial effect on attention levels and may contribute to positive changes in the frontal EEG theta/beta ratio. Most types of yoga positively affect stress reduction in healthy individuals (Soriotulla, 2024) which may have a positive effect on stress-related distraction. It is suggested that yoga practices can be implemented in schools as a preventive measure for children’s mental health problems (Khunti et al., 2023). Yoga helps to reduce stress, improve sleep quality, support emotional and mental health, and reduce symptoms of depression. In addition, yoga improves balance and flexibility and supports strength and endurance (Çetinoğlu and Pehlivan, 2024). It is an expected result that all these benefits will reflect positively on the attention levels of athletes. It is thought that yoga practices will be useful in improving the performance of athletes. It is recommended to include yoga practices as a performance-supporting practice in sports environments.

### 4.1 Comparative discussion of quantitative and qualitative data

The statement of the coach coded C1, “We observed a significant increase in the attention levels of our athletes who participated in kids yoga practices during training and matches. After the practice, we noticed that the athletes acted in a more focused manner, responded better to the strategies in the game, and their distraction decreased. I observed that our athletes were able to remain more patient and concentrated, especially during long-term training or challenging matches, thanks to the mental relaxation brought by yoga” and the statement of coach C2, “It helped them lose less attention during the game and perform better at critical moments” emphasize the positive effects of yoga practices on attention and performance. The statements of the coaches show that kids yoga has positive effects on both mental and physical performance. The qualitative findings obtained within the scope of the study are parallel to the quantitative findings of the study. The findings, supported by both quantitative and qualitative data, are consistent with the literature. While challenging training and matches can create stress on young athletes, yoga helps them to be more patient and focused by reducing this stress. Khalsa et al. (2012) reported that yoga practice increases attention span and mental relaxation. In addition, yoga improves athlete’s performance by reducing anxiety (Weaver and Darragh, 2015). Kauts and Sharma (2009) reported that yoga practice helps students to be more attentive in their classes, which helps athletes make better decisions and reduces distraction. Neurophysiological findings also show that kids yoga is effective in increasing mental and physical awareness (Kabat-Zinn, 2005). In a study of school children (Gulati et al., 2019), the positive effects of yoga on punctuality, concentration and attention skills in children may explain the positive changes in the speed at which athletes read the game and respond strategically on the field. The decrease in theta/beta ratio after yoga practice may have helped athletes develop better attention control and strategic thinking skills during the game. This is especially advantageous in situations where quick decisions and strategic planning are critical.

These findings suggest that kids yoga should be more widely implemented in educational settings. It may be beneficial for educators to use yoga as a tool to increase academic achievement and improve children’s overall well-being. It is also important that education and sports policies encourage the integration of kids yoga (Khunti et al., 2023). In addition, an important limitation of this study is the relatively small sample size (*n* = 20). This may reduce statistical power and limit the generalizability of the findings. Therefore, the results obtained should be interpreted with caution. Future studies with larger and more diverse samples are needed to test the reproducibility of the findings on a larger group of participants and to increase their generalizability. Future research could examine the effects of kids yoga on attention development with larger age groups and samples. These findings will encourage further studies on how kids yoga can be expanded to different sports and age groups, strengthening its potential to improve athletes’ performance and mental health.

### 4.2 Conclusion and recommendations

This study revealed that 8 weeks of kids yoga practice led to a significant increase in the attention levels of volleyball players and that this increase was statistically significant. While the quantitative findings were supported by the increase in the scores obtained from the attention test and the decrease in the frontal EEG theta/beta ratio, the qualitative opinions of the coaches were also consistent with these findings. An inverse relationship was found between stress-induced attention deficit, attention orientation and attention control and theta/beta ratio in healthy individuals (Putman et al., 2014; Morillas-Romero et al., 2015; Angelidis et al., 2016). This inverse relationship is interpreted as the decrease in theta/beta ratio as positive effects develop in attention levels. Within the scope of this study, it can be said that the positive effects of yoga practices on attention levels are supported by the increase in the scores of the athletes in d2 attention tests and the decrease in the frontal theta/beta ratio. This result is consistent with previous studies. These findings were also supported by qualitative findings. The coaches included in the study reported that the volleyball players focused better during training and matches and responded better to strategies. They also emphasized that yoga improves stress coping skills and in-creases mental endurance.

#### 4.2.1 Recommendations

1. Integration of Yoga Programs: Kids yoga practices should be included in athletes’ training programs. Regular yoga sessions are recommended especially for the attention development of young athletes.
2. Education and Awareness: Providing trainings on yoga and mindfulness to coaches and athletes can provide a better understanding of the benefits of these practices.
3. Long-Term Research: It is recommended that studies be conducted to examine the long-term effects of yoga practices by increasing the duration of the practice.
4. Individual Needs: Customized yoga programs should be created according to the individual need of each athlete, and attention development and stress management should be targeted with these programs.
5. Neurophysiological Studies: Studies investigating the neurophysiological effects of kid’s yoga should be continued, and the relationship between EEG measurements and yoga practices should be examined in depth.
6. Sample Size: It is recommended that similar protocols be repeated with larger and more representative samples in future studies.
7. Gender Differences: It is recommended that future studies include both male and female participants to examine possible gender differences and increase the generalizability of the findings.
8. Age Group: It is recommended that participants from a wider age range be included in future studies to assess whether the findings are valid across developmental stages.
9. Sports Branches: It is recommended that future studies be conducted in different sports branches to determine whether the observed effects are specific to volleyball or applicable to other sports branches.
10. Future studies may include task-based EEG protocols simulating volley-ball-specific cognitive and motor demands to better understand the neural correlates of sport-specific performance.

In conclusion, the findings of this study support that kid’s yoga is an effective intervention in increasing the attention levels of athletes. Yoga should be considered as an important tool to improve the mental and physical performance of athletes and should be included in sports training programs.

#### 4.2.2 Limitations

1. An important limitation of this study is the relatively small sample size (*n* = 20).
2. One of the limitations of the study is that the generalizability of the findings is limited due to the inclusion of only female volleyball players.
3. One of the limitations of the study is that the participants were only from the young age group (Mage = 11.45); this limits the generalizability of the results to different age groups.
4. One of the limitations of the study is that only volleyball athletes were included; this limits the generalization of the findings to other sports.
5. Another limitation of this study is that EEG recordings were recorded at rest. Volleyball-specific task-based EEG was not evaluated.

## Data Availability

The raw data supporting the conclusions of this article will be made available by the authors, without undue reservation.

## Footnotes

^1^
https://help.mindmedia.com/en_US/articles/421001-nexus-10-mkii-user-manual (Accessed November 20, 2024).

^2^
https://help.mindmedia.com/en_US/articles/421067-biotrace-software-user-manual (Accessed April 21, 2025).
